# Fine-Tuning Large Language Models for Structured Extraction of Infectious Disease–Related Information From Clinical Notes in Japanese Primary Care: Development and Internal Validation Study

**DOI:** 10.2196/84974

**Published:** 2026-09-10

**Authors:** Hiroshi Yoshihara, Haruka Maeda, Yuriko Hagiwara, Daichi Sato, Kei Kitajima, Akihiro Iwata, Nicolas Van de Velde, Yuta Nakamura, Yosuke Yamagishi, Ataru Igarashi

**Affiliations:** 1 Department of Health Policy and Public Health, Graduate School of Pharmaceutical Sciences, The University of Tokyo, 7-3-1 Hongo, Bunkyo-ku, Tokyo, 113-0033, Japan, 81 3-5841-4828; 2 Department of Respiratory Infections, Institute of Tropical Medicine, Nagasaki University, Nagasaki, Japan; 3 M3 Inc., Tokyo, Japan; 4 Moderna, Inc., Cambridge, MA, United States; 5 Department of Computational Diagnostic Radiology and Preventive Medicine, Graduate School of Medicine, The University of Tokyo, Tokyo, Japan; 6 Division of Radiology and Biomedical Engineering, Graduate School of Medicine, The University of Tokyo, Tokyo, Japan

**Keywords:** natural language processing, large language model, electronic health record, EHR, infectious diseases, public health

## Abstract

**Background:**

The COVID-19 pandemic highlighted the importance of timely infectious disease surveillance. In Japan, conventional sentinel and claims-based systems incur reporting lags and capture limited clinical detail, whereas free-text clinical notes in electronic health records (EHRs) hold richer, timelier symptom and vaccination information. Natural language processing (NLP) with large language models (LLMs) offers a way to structure such free text at scale.

**Objective:**

We aimed to develop and internally validate an NLP algorithm to extract structured infectious disease–related symptoms and vaccination history from free-text clinical notes in Japanese primary care, as a feasibility step toward low-latency, EHR-based surveillance.

**Methods:**

A total of 773 clinical notes, originating from 526 unique patients, were provided by M3 Inc through the Japan Medical Data Survey and used for analysis. Three physicians annotated information related to infectious disease symptoms and vaccination history. The data were divided into 622 (80%) training cases and 151 (20%) evaluation cases with no patient overlap. We compared a physician-designed, rule-based algorithm, few-shot learning (FSL) using commercial and open-source LLMs, and supervised fine-tuning (SFT) of open-source LLMs, using the macroaveraged *F*_1_-score (unweighted mean across 9 clinical categories). Sensitivity, specificity, positive predictive value (PPV), and negative predictive value (NPV) were also computed, with 95% CIs from a patient-level cluster bootstrap (2000 replicates).

**Results:**

Rule-based extraction achieved a macroaveraged *F*_1_-score of 0.685 (95% CI 0.630-0.736). FSL markedly improved the extraction of high-variability items such as vaccination history and onset date. Anthropic Claude 3.5 Sonnet achieved a macroaveraged *F*_1_-score of 0.875 (95% CI 0.800-0.913; sensitivity 0.929, specificity 0.918). SFT of Google’s open-source Gemma 2 27B model with quantized low-rank adaptation (QLoRA) achieved the highest point estimate (macroaveraged *F*_1_-score of 0.906, 95% CI 0.833-0.945; sensitivity 0.921, specificity 0.969, PPV 0.906); the difference from Claude 3.5 Sonnet was small and not statistically distinguishable (Δ*F*_1_-score=0.030, 95% CI −0.035 to 0.140). A small, fine-tuned Gemma 2 2B model reached 0.822 (95% CI 0.752-0.874), significantly lower than that of the 27B model (Δ*F*_1_-score=0.084, 95% CI 0.040-0.163).

**Conclusions:**

A fine-tuned open-source LLM can accurately extract and structure infectious disease–related information from Japanese free-text clinical notes, achieving performance comparable to that of a commercial model while enabling processing within a closed environment. These findings support the feasibility of EHR-based digital surveillance, whose downstream utility remains to be demonstrated.

## Introduction

The COVID-19 pandemic underscored the importance of timely infectious disease surveillance, and this need has persisted in the postpandemic era. Influenza, COVID-19, and respiratory syncytial virus (RSV) now cocirculate, present overlapping symptoms, and can cause simultaneous outbreaks that strain health care systems [1-3]. Surveillance systems that can rapidly detect and characterize such activity therefore remain a public health priority.

In Japan, harnessing electronic health record (EHR) data has the potential to offer particular advantages for infectious disease surveillance over traditional claims data. The country’s conventional surveillance systems, such as the national sentinel provider network for influenza, typically aggregate case reports on a weekly basis and often incur reporting lags of approximately 1 week [4]. This delay can slow down outbreak detection. To achieve lower latency, researchers have turned to alternative data sources such as health insurance claims and pharmacy records. Notably, a real-time prescription surveillance system introduced in Japan demonstrated that daily tracking of antiviral drug prescriptions could closely estimate influenza case numbers in near real time [4]. However, although claims and prescription data provide faster updates, they contain only limited clinical details, primarily billing codes for diagnoses or medications, and may not capture the nuanced symptom information available in a clinical note. Claims-based metrics also risk being biased by changing coding practices and may lack clinical fidelity [5]. By contrast, EHR clinical notes contain rich descriptions of patient symptoms, findings, and vaccination history recorded at the point of care. These narratives can be accessed soon after the medical encounter, giving a timelier and more detailed picture of emerging infections. Studies have found that surveillance based on EHR-derived clinical data can provide more objective and reliable estimates of disease incidence than surveillance based on claims data [5].

Natural language processing (NLP) and deep learning are promising tools to address some of these surveillance challenges. NLP is a branch of AI that focuses on enabling computers to understand and generate human language [6]. Recently, deep learning techniques have advanced NLP capabilities, allowing automated analysis of large volumes of text. This is especially significant in health care, where enormous amounts of data are recorded as unstructured text in EHRs [6]. Applications of NLP in health care are broad: systems have been developed to classify medical notes by content, recognize and extract clinical entities (such as symptoms, diagnoses, or medications) from text, summarize patient records, and even translate medical jargon into lay language [7]. Recent large language models (LLMs) are used to parse free-text EHR narratives and extract structured clinical concepts with unprecedented accuracy. Many studies have concluded that commercial and open-source LLMs could outperform traditional NLP systems in extracting various types of information from medical notes [8,9].

Internationally, numerous studies in the Netherlands [10], the United States [11-13], Singapore [14], and New Zealand [15] have used NLP to identify infectious disease cases or to extract related clinical information from EHRs for case detection and surveillance. In Japan, however, epidemiologists have relied mainly on structured data, such as outpatient prescription records [16], and, to our knowledge, no study has directly extracted infectious disease information from unstructured Japanese clinical narratives. In this study, we aim to address this gap by developing and validating an NLP algorithm that leverages state-of-the-art LLMs to extract infectious disease–related symptoms and vaccination history from free-text clinical notes written in Japanese, as a feasibility step toward timelier, EHR-based digital surveillance.

## Methods

### Data Source and Study Population

A pseudonymized extract of clinical notes from the Japan Medical Data Survey (JAMDAS; M3 Inc), an outpatient health care database of nationwide primary care clinics curated by M3 Inc [17], was used for analysis. The 5 outpatient clinics were a convenience sample of facilities participating in the database; they were not randomly drawn from JAMDAS, and patients within these clinics were randomly sampled with no filtering by disease name or prescription. The dataset therefore includes patients with a variety of medical conditions. It comprised 2020 clinical notes from 1036 patients aged 0 to 84 years who visited these clinics for fever or suspected infectious disease between October 2020 and September 2023. The clinics served distinct populations (median patient age ranging from approximately 1 year at a pediatric-predominant clinic to approximately 37 years at an adult clinic; Table S6 in Multimedia Appendix 1).

### Annotation and Interrater Agreement

After removing missing values and duplicate records, 3 physicians within the research team performed annotations on the following items: body temperature, fatigue, muscle and joint pain, headache and dizziness, respiratory symptoms, visible changes in the throat, gastrointestinal symptoms, sensory abnormalities, other symptoms, vaccination information, and onset date. Detailed annotation items, criteria, and extraction formats are described in Table 1. The raw onset description, parsed onset date, body temperature, and description of vaccination history were annotated as text data types, while all other items were classified as binary variables.

**Table 1.** Dataset characteristics and extraction item definitions^a^.

Items	Training dataset (n=622)	Evaluation dataset (n=151)	*P* value	Definition	Format	Example
Demographic information						
Age (years), mean (SD)	16.2 (15.7)	18.3 (16.8)	.16	—^b^	—	—
Male sex, n (%)	350 (56.3)	70 (46.4)	.04	—	—	—
Symptom onset, n (%)						
Parsed date mentioned	496 (79.7)	119 (78.8)	.89	Date of initial symptom onset determined from the visit date and clinical note description	Text	2022-10-15
Raw description mentioned	503 (80.9)	121 (80.1)	.93	Raw description of the initial symptom onset date in the clinical note	Text	昨日朝 (English: yesterday morning)
Body temperature, n (%)						
Body temperature mentioned	420 (67.5)	99 (65.6)	.72	Highest body temperature recorded in the clinical note. Annotations were standardized to the format “3x.x”	Text	39.4
Slight fever	78 (12.5)	18 (11.9)	.94	Whether slight fever is mentioned in the clinical note or the patient’s highest body temperature is between 37 °C and 37.4 °C	Binary	1
Fever	369 (59.3)	89 (58.9)	1	Whether fever is mentioned in the clinical note or the patient’s highest body temperature is ≥37.0 °C	Binary	0
High fever	190 (30.5)	47 (31.1)	.97	Whether high fever is mentioned in the clinical note or patient’s highest body temperature is ≥38 °C	Binary	1
Chills	12 (1.9)	3 (2)	1	Presence of the symptom	Binary	0
Fatigue and muscle or joint pain, n (%)						
Fatigue	89 (14.3)	21 (13.9)	1	Presence of the symptom	Binary	1
Muscle or joint pain	18 (2.9)	5 (3.3)	.997	Presence of the symptom	Binary	0
Headache and dizziness, n (%)						
Dizziness	3 (0.5)	1 (0.7)	1	Presence of the symptom	Binary	1
Headache	139 (22.3)	33 (21.9)	.98	Presence of the symptom	Binary	0
Respiratory symptoms, n (%)						
Runny nose	222 (35.7)	54 (35.8)	1	Presence of the symptom	Binary	1
Nasal congestion	17 (2.7)	4 (2.6)	1	Presence of the symptom	Binary	0
Sore throat	239 (38.4)	57 (37.7)	.95	Including discomfort in the throat	Binary	1
Cough	275 (44.2)	68 (45)	.93	Presence of the symptom	Binary	0
Sputum	104 (16.7)	24 (15.9)	.90	Presence of the symptom	Binary	1
Chest pain	3 (0.5)	0 (0)	.90	Presence of the symptom	Binary	0
Shortness of breath	3 (0.5)	0 (0)	.90	Presence of the symptom	Binary	1
Breathing sounds	11 (1.8)	3 (2)	1	Presence of abnormal breath sounds based solely on objective physical examination findings (excluding patient-reported symptoms)	Binary	0
Throat appearance, n (%)						
Tonsillar hypertrophy	1 (0.2)	0 (0)	1	Presence of the symptom	Binary	1
Tonsillar exudates	3 (0.5)	1 (0.7)	1	Presence of the symptom	Binary	0
Pharyngeal redness	113 (18.2)	27 (17.9)	1	Including redness of the tonsils	Binary	1
Gastrointestinal symptoms, n (%)						
Abdominal pain	22 (3.5)	5 (3.3)	1	Presence of the symptom	Binary	1
Diarrhea	28 (4.5)	7 (4.6)	1	Presence of the symptom	Binary	0
Nausea	21 (3.4)	5 (3.3)	1	Presence of the symptom	Binary	1
Vomiting	21 (3.4)	5 (3.3)	1	Presence of the symptom	Binary	0
Other symptoms, n (%)						
Taste abnormality	4 (0.6)	0 (0)	.72	Presence of the symptom	Binary	1
Smell abnormality	1 (0.2)	1 (0.7)	.84	Presence of the symptom	Binary	0
Lymph node swelling	3 (0.5)	1 (0.7)	1	Including swelling of all lymph nodes, regardless of location	Binary	1
Rash	11 (1.8)	3 (2)	1	Presence of rash or skin eruption	Binary	0
Vaccination information, n (%)						
COVID-19 vaccination	141 (22.7)	34 (22.5)	1	Presence of COVID-19 vaccination, regardless of the number of doses	Binary	1
Raw description mentioned	194 (31.2)	47 (31.1)	1	Descriptions related to vaccines (including those other than COVID-19)	Text	コロナワクチン接種：2回, 最終2021年10月16日,Pfizer (English: COVID-19 vaccination: 2 doses, most recent on October 16, 2021, Pfizer)

a Data are from a development and internal validation study of large language models for extracting infectious disease–related information from free-text clinical notes of Japanese primary care outpatients in the Japan Medical Data Survey database between October 2020 and September 2023. The table shows patient demographics and the distribution of each extraction item in the training (n=622) and evaluation (n=151) datasets, together with each item’s annotation criteria, extraction format, and worked examples. Binary items are summarized as n (%) notes in which the item was mentioned, and continuous variables (eg, body temperature) are summarized by their distribution. *P* values compare the training and evaluation sets.

b Not applicable.

To assess interrater reliability, a pilot set of 216 notes was independently annotated by all 3 physicians; the remaining notes were annotated by a single physician. In the pilot set, the mean observed agreement for binary symptom flags was 93.9% (unanimous, 3/3, 91.1%), with a pooled Fleiss κ of 0.31; the low κ reflects the very low prevalence of most symptoms (the κ paradox) rather than poor agreement. For the fever flag, the physicians’ operational definitions initially diverged when no temperature had been measured (κ≈0.02); this was resolved by consensus into a single harmonized definition that was applied to the finalized reference standard. For free-text and numeric items, exact-match agreement among all 3 physicians was 71.8% for body temperature and 99.5% for vaccination descriptions (per-item Fleiss κ and observed agreement are given in Table S4 in Multimedia Appendix 1). Disagreements were resolved by majority vote (≥2 of 3 physicians). Of the 151 evaluation notes, 45 (30%) were triple annotated and 106 (70%) were single annotated; a sensitivity analysis showed comparable extraction performance between the 2 subsets (Table S8 in Multimedia Appendix 1).

Owing to the cost of manual annotation, only a portion of the 2020 candidate notes could be annotated: 802 (40%) notes (526 patients) were annotated, and 773 (96%) of these, after removing notes with missing or duplicated diagnostic free text, formed the analysis set. The remaining unannotated candidate notes were therefore excluded because of labeling resource constraints and not because the annotators failed to reach consensus.

### Algorithm Development

The dataset was randomly divided into 622 (80%) training cases and 151 (20%) evaluation cases, ensuring no patient overlap between the two sets. We developed and compared the following approaches: a rule-based algorithm crafted by physicians; few-shot learning (FSL) using commercial and open-source LLMs; and fine-tuning of open-source LLMs. The rule-based algorithm was developed by physicians who carefully reviewed the training dataset and translated patterns for each extraction item into regular expressions.

After several preliminary performance experiments, this study selected Claude 3.5 Sonnet (version 20241022; Anthropic PBC) [18] as the representative commercial LLM and the Gemma 2 family (27B and 2B models; Alphabet LLC) [19] as the representative open-source LLMs. We tested several prompt patterns and settled on one that outputs results in a JSON format, where each extraction item serves as a key and the corresponding extracted value serves as its value (eg*, {“fever”: 1, “temperature”: 38.3, “headache”: 0}*). The prompt used is shown in Textbox 1.

Textbox 1. **Large language model prompts used for structured data extraction.**

**Prompt for few-shot learning**

System: Carefully analyze the following instruction, drawing upon your extensive knowledge and relevant references.

Keep the answer short and do not provide explanations or notes.

# Instructions:

Your task is to extract items from a medical record in the context and return the results as JSON strings.

If you don’t find an item in the context or you are not sure, skip the item.

# Format:

Return JSON strings ONLY in a single line.

Output a single JSON with multiple keys.

JSON key must be one of the items below, do NOT change the item name.

JSON value is the extracted number or text.

Remove units such as kg.

# Items:

{description of extraction items}

For binary items, negative statement = =0, positive statement = =1.

# Examples:

{multiple examples}

# Now please extract items from the context below.

Context: {clinical note to process}

Answer:

**Prompt for fine-tuning**

# Instructions:

Your task is to extract items from a medical record in the context and return the results as JSON strings.

If you don’t find an item in the context or you are not sure, skip the item.

# Format:

Return JSON strings ONLY in a single line.

Output a single JSON with multiple keys.

JSON key must be one of the items below, do NOT change the item name.

Do NOT change the order of items.

JSON value is the extracted number or text.

Remove units such as kg.

# Items:

{description of extraction items}

For binary items, negative statement = 0, positive statement = 1.

# Examples:

{single example}

# Now please extract items from the context below.

Context:{clinical note to process}

Answer:

Within each of 9 clinical categories, per-note item observations were pooled (microaveraged) to compute *F*_1_-score; the overall value is the unweighted macroaverage across categories. Each cell shows the *F*_1_-score point estimate with its 95% CI from a patient-level cluster bootstrap (2000 replicates). Categories with no positive cases were undefined and excluded from the macroaverage.

FSL is a technique in which a model is given a small number of example inputs and outputs to learn from before performing a task [20]. This allows the model to better understand the task and generate more accurate results without updating its parameters. We initially planned to compare zero-shot inference with FSL inference. However, we found that zero-shot outputs often did not adhere strictly to the JSON format, making reliable evaluation difficult. Therefore, we used the FSL results as the baseline for LLM performance. We randomly sampled 18 examples, ensuring that each extraction item had at least 1 positive instance included.

To further improve performance, we applied supervised fine-tuning to the open-source LLMs. The training dataset was split into 500 (80%) samples for training and 122 (20%) samples for validation, with the validation set used to monitor overfitting during training. We performed fine-tuning using the instruction-tuned Google Gemma 2 models (gemma-2-2b-it and gemma-2-27b-it). Full fine-tuning was conducted on the 2B model, while, owing to hardware limitations, we applied quantized low-rank adaptation (QLoRA) for fine-tuning the 27B model. Low-rank adaptation (LoRA) is a technique that reduces the number of trainable parameters by injecting low-rank matrices into a pretrained model’s weights, enabling efficient fine-tuning without updating the entire model [21]. QLoRA combines LoRA with quantization, compressing model weights to reduce memory use while maintaining accuracy, allowing large models to be fine-tuned on limited hardware resources [22]. The specific training parameters are detailed in Table S1 in Multimedia Appendix 1. Fine-tuning was performed using the transformers [23] and trl [24] libraries on a single RTX 6000 Ada GPU (NVIDIA Corp).

### Evaluation

We grouped the extraction items into 9 clinical categories and computed performance within each category. Each extracted item in a note was treated as 1 binary observation; for items extracted as text or numeric values (eg, body temperature, onset date, and vaccination description), an exact-value match was required. Within each category, per-note item observations were pooled (microaveraging) to compute the *F*_1_-score, sensitivity, specificity, positive predictive value (PPV), and negative predictive value (NPV); the overall value for each metric was the unweighted macroaverage across the 9 categories, which served as the primary performance metric. Categories with no positive cases in the evaluation set were undefined and were excluded from the average. Metrics were thus computed at the level of item presence within a note, pooled within a category and then macroaveraged across categories—not aggregated at the patient or note level. Moreover, 95% CIs were obtained by patient-level cluster bootstrap resampling with 2000 replicates; and paired between-model differences were assessed with the same patient-level bootstrap. Per-category sensitivity, specificity, PPV, and NPV are provided in Table S3 in Multimedia Appendix 1.

### Ethical Considerations

This study was approved by the Nihonbashi-Sakura Clinic Ethics Committee. The committee did not issue an approval or case number for this study, so none is reported. Because the analysis used pseudonymized, retrospective data recorded during routine clinical care, individual informed consent was not obtained; instead, in accordance with the Japanese Ethical Guidelines for Medical and Biological Research Involving Human Subjects, information about the study was disclosed to patients and an opt-out opportunity to decline participation was provided. All records were pseudonymized before they were made available to the research team, and the data were accessed and handled solely in accordance with the restricted-use data agreement with M3 Inc that governs the JAMDAS database. The study was conducted in accordance with the Declaration of Helsinki.

## Results

### Overview

**Figure 1.**
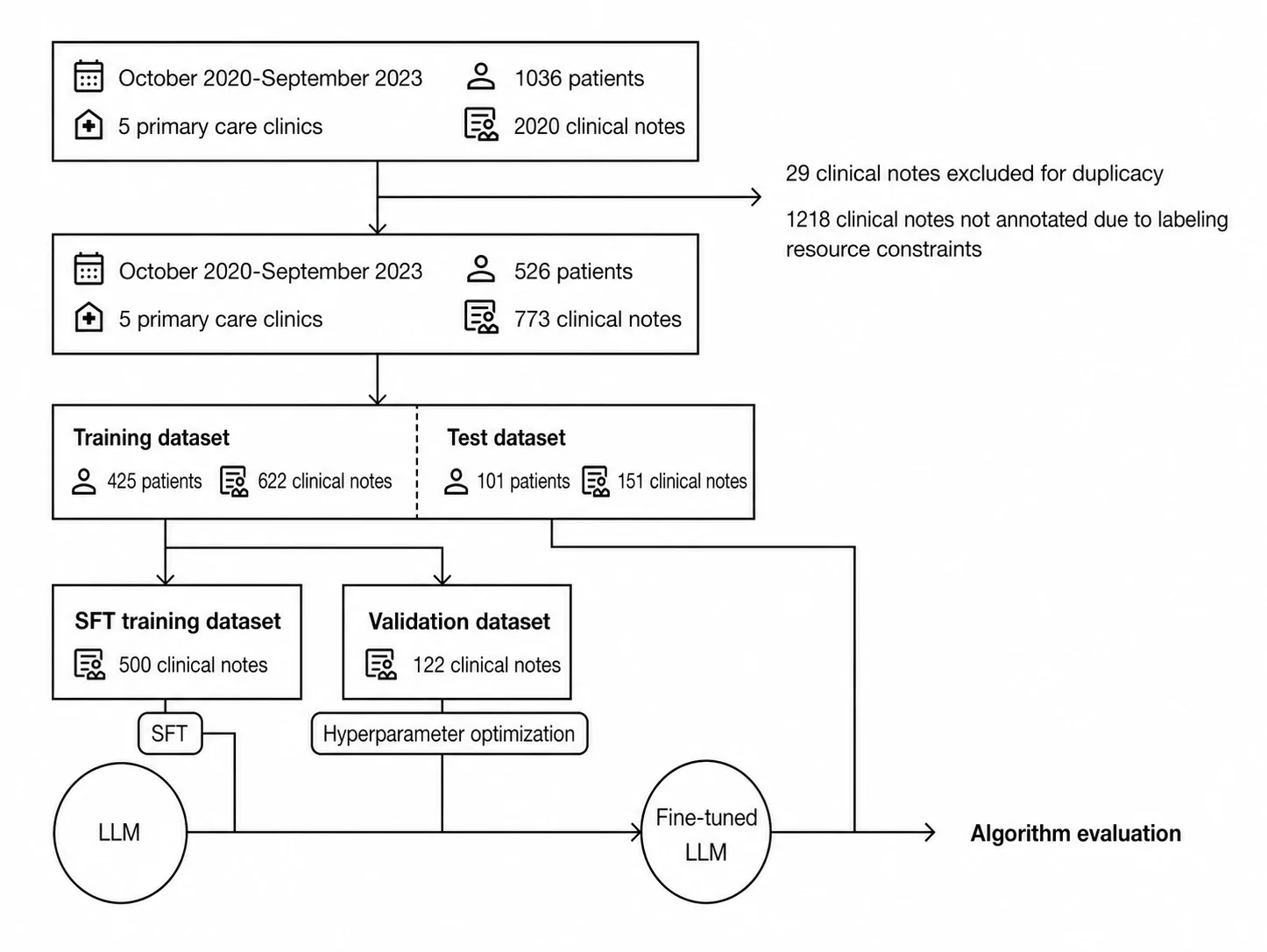
Data flowchart and study overview. LLM: large language model; SFT: supervised fine-tuning.

Of the 2020 candidate notes (1036 patients), 802 (526 patients) were annotated; the remainder were left unannotated owing to labeling resource constraints. After removing notes with missing or duplicated diagnostic free text, 773 notes from 526 patients formed the analysis set. Compared with the annotated notes, the unannotated notes came from patients who were significantly younger (median age 3, IQR 2-17 vs 15, IQR 7-20 years; *P*<.001) and were drawn disproportionately from particular clinics (*P*<.001); sex did not differ (*P*=.44; Table S5 in Multimedia Appendix 1). The data flow and an overview of the experiments are shown in Figure 1. Background information and the distribution of extraction items for the training and evaluation datasets are presented in Table 1.

The diagram shows the flow from 2020 candidate notes (1036 patients) through annotation (802 notes; 526 patients) to the analysis set (773 notes; 526 patients), and the random, patient-disjoint split into a training set (622 notes; 500 training and 122 validation) and a held-out evaluation set (151 notes) used to compare the rule-based, few-shot, and fine-tuned approaches.

Interrater reliability was assessed on a 216-note pilot set independently annotated by all 3 physicians; the remaining notes, including 106 (70%) of the 151 evaluation notes, were single annotated.

As shown in Table S2 in Multimedia Appendix 1, although there were some systematic differences in clinical note templates across clinics and physician preferences within clinics, the notes were generally written in the SOAP (subjective, objective, assessment, and plan) format, showing overall similarity. The performance evaluation results for each model are presented in Table 2. Some extraction items exhibited relatively low variability in their descriptions, indicating that their descriptions were similar across many physicians; consequently, the rule-based algorithm achieved high performance on these items. For items with high variability in descriptions, such as vaccination information and onset dates, the rule-based algorithm showed low accuracy; however, FSL with LLMs achieved high accuracy. In particular, the commercial model Anthropic Claude 3.5 Sonnet achieved a macroaverage *F*_1_-score of 0.875 (95% CI 0.800-0.913), with a sensitivity of 0.929.

**Table 2.** Extraction performance by category and algorithm^a^.

Categories	Rule-based, *F*_1_-score (95% CI)	Gemma 2 2B+FSL^b^, *F*_1_-score (95% CI)	Gemma 2 27B+FSL, *F*_1_-score (95% CI)	Claude 3.5 Sonnet+FSL, *F*_1_-score (95% CI)	Gemma 2 2B+SFT^c^, *F*_1_-score (95% CI)	Gemma 2 27B+quantized low-rank adaptation SFT, *F*_1_-score (95% CI)
Body temperature	0.855 (0.815-0.893)	0.671 (0.624-0.713)	0.864 (0.831-0.896)	0.899 (0.868-0.928)	0.863 (0.828-0.897)	0.944 (0.914-0.968)
Fatigue and muscle or joint pain	0.926 (0.846-0.984)	0.744 (0.564-0.884)	0.863 (0.735-0.958)	0.945 (0.880-1.000)	0.824 (0.682-0.931)	0.926 (0.846-0.984)
Headache and dizziness	0.879 (0.784-0.955)	0.757 (0.64-0.853)	0.93 (0.862-0.985)	0.93 (0.862-0.985)	0.93 (0.862-0.985)	0.93 (0.862-0.985)
Respiratory symptoms	0.892 (0.849-0.928)	0.672 (0.607-0.73)	0.925 (0.888-0.955)	0.936 (0.9-0.966)	0.868 (0.825-0.903)	0.938 (0.904-0.967)
Throat appearance	0.731 (0.556-0.861)	0.667 (0.489-0.800)	0.862 (0.750-0.944)	0.824 (0.706-0.914)	0.915 (0.833-0.979)	0.915 (0.824-0.980)
Gastrointestinal symptoms	0.746 (0.588-0.857)	0.85 (0.722-0.950)	0.955 (0.857-1.000)	0.894 (0.780-0.982)	0.837 (0.684-0.957)	0.955 (0.878-1.000)
Other symptoms	0.533 (0.213-0.815)	0 (0-0)	0.750 (0-1.000)	0.800 (0-1.000)	0.500 (0-0.800)	0.750 (0-1.000)
Vaccination information	0.437 (0.324-0.528)	0.327 (0.208-0.437)	0.699 (0.582-0.793)	0.755 (0.657-0.837)	0.776 (0.674-0.863)	0.836 (0.750-0.908)
Onset date	0.168 (0.111-0.22)	0.827 (0.78-0.868)	0.923 (0.891-0.952)	0.895 (0.857-0.929)	0.887 (0.847-0.923)	0.957 (0.934-0.978)
Overall (macroaverage)	0.685 (0.630-0.736)	0.613 (0.570-0.653)	0.863 (0.797-0.902)	0.875 (0.800-0.913)	0.822 (0.752-0.874)	0.906 (0.833-0.945)

a Data are from the held-out evaluation set (n=151).

b FSL: few-shot learning.

c SFT: supervised fine-tuning.

In the fine-tuning experiments, both the Google Gemma 2 2B and 27B models showed substantial performance improvements. The 2B model achieved a macroaveraged *F*_1_-score of 0.822 (95% CI 0.752-0.874), while the 27B model fine-tuned with QLoRA reached the highest point estimate (0.906, 95% CI 0.833-0.945; specificity of 0.969 and PPV of 0.906). The fine-tuned 27B model’s advantage over Claude 3.5 Sonnet was small, and its CI included zero (Δ*F*_1_-score=0.030, 95% CI −0.035 to 0.140); therefore, the 2 models were not statistically comparable. The 27B model did, however, significantly outperform the smaller fine-tuned 2B model (Δ*F*_1_-score=0.084, 95% CI 0.040 to 0.163). Per-category sensitivity, specificity, PPV, and NPV for all models are provided in Table S3 in Multimedia Appendix 1.

### Performance by Primary Diagnosis

Because the cohort was dominated by COVID-19 (approximately 80% of notes mentioned COVID-19), a fine-grained diagnosis breakdown was not feasible; we therefore compared COVID-19 (n=120, 79%) and non–COVID-19 (n=31, 21%) notes in the test set. The best fine-tuned model was stable across strata (macroaveraged *F*_1_-score 0.900 vs 0.893), whereas the rule-based algorithm (0.702 vs 0.605) and Claude 3.5 Sonnet (0.886 vs 0.735) showed lower performance on non–COVID-19 notes. Given the small non–COVID-19 subgroup, these results are exploratory (Table S7 in Multimedia Appendix 1).

### Error Analysis

A qualitative error analysis of misclassifications by the LLM revealed that many errors occurred when multiple extraction targets were listed consecutively, separated by commas or periods, followed by a collective negation (eg, “Diarrhea, nausea negative.” and “Abdominal pain and vomiting. Wheezing negative.”). These cases can be interpreted in multiple ways and are examples of situations in which even human experts would find it difficult to reach a consensus.

## Discussion

### Principal Findings

We demonstrated that LLMs can extract infectious disease–related information and vaccination data from free-text clinical notes written in Japanese in EHRs with high accuracy. For items with high descriptive variability, LLMs markedly outperformed rule-based regular expressions. The commercial model Claude 3.5 Sonnet (with FSL) reached a macroaveraged *F*_1_-score of 0.875 (95% CI 0.800-0.913) without additional training, while fine-tuning the open-source Gemma 2 27B model with QLoRA on only 500 notes achieved a comparable point estimate (0.906, 95% CI 0.833-0.945); the difference between the 2 was small and not statistically distinguishable (Δ*F*_1_-score=0.030, 95% CI −0.035 to 0.140). The fine-tuned 27B model did, however, significantly outperform the smaller fine-tuned 2B model (Δ*F*_1_-score=0.084, 95% CI 0.040 to 0.163). Given the security risks of sending clinical text containing sensitive personal information to external commercial models, achieving comparable performance with a smaller open-source model demonstrates the feasibility of processing data securely within a closed environment, enhancing real-world applicability.

### Comparison With Prior Work and Interpretation of Findings

Our findings are consistent with prior reports that LLMs outperform traditional NLP approaches for structured extraction from EHR free text [8,9,25] and extend them to Japanese primary care narratives, which, to our knowledge, have not previously been targeted directly; prior Japanese clinical NLP has largely used BERT (Bidirectional Encoder Representations from Transformers)-based models or symptom taggers on other document types [26,27]. In contrast to BERT-based COVID-19 case detection in Dutch general practice [10] and rule or curation pipelines in large US EHR systems [11], we show that a compact, locally deployable, fine-tuned generative model can perform comparably to a commercial model, consistent with recent reports that fine-tuned open-source LLMs reach human-level extraction performance from few examples [28] and that local open-source deployment mitigates the privacy risks of sending notes to external services [29], while benchmark studies find that fine-tuning still tends to outperform zero or few-shot prompting for extraction tasks [30].

Several categories scored low under FSL (eg, other symptoms and vaccination information for Gemma 2 2B). This likely reflects (1) class imbalance—rare symptoms such as chills, dizziness, and rash had very few positive cases; (2) ambiguity in clinical expressions, including consecutively listed findings followed by a collective negation—a well-known challenge for negation or assertion detection in clinical text [31,32]; and (3) the limited capacity of small models without parameter updates. Fine-tuning substantially mitigated these issues. The low interrater agreement observed for some common items (eg, fever), which is consistent with prior reports that annotation of clinical narratives is intrinsically variable [33], further indicates that part of the residual error stems from intrinsic annotation ambiguity rather than model failure.

At this feasibility stage, the extraction is fully automated: the structured outputs are intended for subsequent review and aggregation by public health or clinical staff rather than for autonomous clinical decision-making, and no specialized user interaction with the model is required beyond the domain expertise needed to interpret aggregated surveillance signals.

### Limitations

This study has several limitations. First, the 5 clinics were a convenience sample, and the annotated (analyzed) set overrepresented some clinics while leaving others almost entirely unannotated (eg, 2 clinics contributed almost no analyzable notes); the algorithm’s accuracy may not generalize to clinics with different documentation styles. Second, because manual annotation was resource-limited, only approximately 40% of the candidate notes (802 of 2020) were annotated, and these differed systematically from the unannotated notes: annotated patients were older (median age 15 vs 3 years; *P*<.001) and came disproportionately from particular clinics (*P*<.001), although sex did not differ (*P*=.44). Because the choice of which notes to annotate was not random, this raises the possibility of selection bias. Third, the evaluation set was predominantly single annotated (106/151, 70% of notes), so interrater reliability was quantified on the 216-note pilot subset; a sensitivity analysis showed comparable extraction performance between triple- and single-annotated notes (Table S8 in Multimedia Appendix 1). Fourth, we could not establish an external validation set, so the results represent internal validation only. Finally, most patients had a single note, so the ability to capture temporal symptom changes is unverified. Critically, this study demonstrates extraction accuracy, not downstream surveillance utility: we did not evaluate whether the extracted signals improve outbreak detection, trend monitoring, or timeliness in real-world settings.

Future research should (1) validate the algorithm on external clinics not involved in training, (2) link extracted symptom and vaccination signals to observed infectious disease trends to assess surveillance timeliness and accuracy, and (3) extend annotation to longitudinal records.

### Conclusions

By fine-tuning the open-source LLM Google Gemma 2, we developed and internally validated a model specialized in extracting and structuring infectious disease–related information from Japanese free-text clinical notes with accuracy comparable to that of a commercial model, while remaining deployable in a closed environment. Because the model can run locally without transmitting sensitive clinical text to external services, it offers a practical and privacy-preserving foundation for EHR-based digital surveillance in settings where data cannot leave the institution. Realizing that potential will require external validation and, above all, demonstrating that the extracted signals improve real-world outbreak detection and timeliness—the essential next step toward broader public health impact.

## Supplementary material

10.2196/84974 Multimedia Appendix 1 Hyperparameters, note templates, per-category sensitivity/specificity/positive predictive value/negative predictive value with 95% CIs, interrater agreement, annotated-vs-unannotated and per-clinic characteristics, and performance by diagnosis and by annotation depth.

## Abbreviations

BERT: Bidirectional Encoder Representations from Transformers
EHR: electronic health record
FSL: few-shot learning
JAMDAS: Japan Medical Data Survey
LLM: large language model
LoRA: low-rank adaptation
NLP: natural language processing
NPV: negative predictive value
PPV: positive predictive value
QLoRA: quantized low-rank adaptation
RSV: respiratory syncytial virus
SOAP: subjective, objective, assessment, and plan
