# Supplementary material for: Fine-Tuning Large Language Models for Structured Extraction of Infectious Disease–Related Information From Clinical Notes in Japanese Primary Care: Development and Internal Validation Study
Source: JMIR Form Res. 2026 Sep 10;10:e84974. doi: 10.2196/84974 (PMC13561044; doi:10.2196/84974)
Supplement: Supplementary file 1 [file formative-v10-e84974-s001.docx]

## Supplementary Table 1. Parameters used for model training

| Parameter | Value |
| --- | --- |
| Gemma 2 2B full fine-tuning | |
| Optimizer | paged_adamw_8bit |
| Scheduler | cosine |
| Learning rate | 4.00E-06 |
| Warmup ratio | 0.1 |
| Batch size | 2 |
| Gradient accumulation | 8 |
| Train epochs | 5 |
| Gradient checkpointing | TRUE |
| Max sequence length | 2048 |
| Dataset packing | TRUE |
| Gemma 2 27B QLoRA | |
| Optimizer | paged_adamw_8bit |
| Scheduler | cosine |
| Learning rate | 1.00E-04 |
| Warmup ratio | 0.1 |
| Batch size | 2 |
| Gradient accumulation | 8 |
| Train epochs | 5 |
| Gradient checkpointing | TRUE |
| Max sequence length | 2048 |
| Dataset packing | TRUE |
| LoRA modules | ['q_proj', 'k_proj', 'v_proj', 'o_proj', 'gate_proj', 'up_proj', 'down_proj'] |
| LoRA r | 64 |
| LoRA alpha | 64 |
| LoRA dropout | 0.05 |
| Quantization | BnB 4bit |

## Supplementary Table 2. Examples of clinical notes

| Clinic ID | Original clinical note | English translation |
| --- | --- | --- |
| 1 | 【名前】 XXX  【生年月日】 XXX  【性別】 XXX  【住所】 XXX  【電話番号】 XXX  【どのような症状が出ていますか？】 XXX  【いつから症状がでましたか？】 XXX  【検査を希望しますか？】 XXX  【自宅や無料検査場でPCR検査または抗原検査を実施し陽性でしたか？】 XXX  【新型コロナウイルス陽性者との濃厚接触のエピソードはありますか？】 XXX  【処方を希望しますか？】 XXX  【既往歴】 XXX  【妊娠していますか？】 XXX  【所見】 XXX | [Name] XXX  [Date of Birth] XXX  [Sex] XXX  [Address] XXX  [Phone Number] XXX  [What symptoms are you experiencing?] XXX  [When did the symptoms start?] XXX  [Do you wish to undergo testing?] XXX  [Have you tested positive with a PCR or antigen test at home or a free testing site?] XXX  [Any episode of close contact with a confirmed COVID-19 positive case?] XXX  [Do you wish to receive a prescription?] XXX  [Past Medical History] XXX  [Are you pregnant?] XXX  [Findings] XXX |
| 2 | 【主訴】  XXX  【現病歴】  XXX  【既往歴】  XXX  【アレルギー】  XXX  【内服薬】  XXX  【身体所見】  XXX  【重症リスク】  XXX  【検査】  XXX  【A/P】  XXX | [Chief Complaint]  XXX  [Present Illness]  XXX  [Past Medical History]  XXX  [Allergies]  XXX  [Current Medications]  XXX  [Physical Findings]  XXX  [Risk Factors for Severe Disease]  XXX  [Examinations]  XXX  [A/P]  XXX |
| 3 | 体温 XXX ℃  体重 XXX kg  再診  XXX  S) XXX  O: general status XXX  XXX  A) XXX  P) XXX | Body Temperature XXX ℃  Body Weight XXX kg  Follow-up Visit  XXX  S) XXX  O: general status XXX  XXX  A) XXX  P) XXX |
| 4 | 発熱・風邪用  S)【現病歴】  有症状：XXX  いつから：XXX  Sick contact：XXX  海外渡航歴：XXX  濃厚接触：XXX  コロナワクチン接種：XXX  【既往歴】  XXX  新型コロナウイルス感染歴：XXX  【生活歴】  喫煙：XXX  飲酒：XXX  職業：XXX  ADL・家族：XXX  【アレルギー】  XXX  【内服】  XXX  O) XXX  A/P)  XXX | For Fever / Cold  S) [Present Illness]  Symptoms: XXX  Onset: XXX  Sick contact: XXX  Overseas travel history: XXX  Close contact: XXX  COVID-19 vaccination: XXX  [Past Medical History]  XXX  History of COVID-19 infection: XXX  [Social History]  Smoking: XXX  Alcohol: XXX  Occupation: XXX  ADL / Family: XXX  [Allergies]  XXX  [Current Medications]  XXX  O) XXX  A/P)  XXX |
| 5 | 体温 XXX ℃  S: XXX  O: XXX  A: XXX  P: XXX | Body Temperature XXX ℃  S: XXX  O: XXX  A: XXX  P: XXX |

All descriptions related to actual patients have been masked with XXX.

## Supplementary Table S3. Sensitivity, specificity, PPV, and NPV for all models

| **Model** | **Sensitivity** | **Specificity** | **PPV** | **NPV** |
| --- | --- | --- | --- | --- |
| Rule-based | 0.732 (0.687–0.778) | **0.968 (0.957–0.977)** | 0.756 (0.695–0.826) | 0.880 (0.868–0.892) |
| Gemma 2 2B + FSL | 0.562 (0.516–0.616) | 0.917 (0.894–0.938) | 0.692 (0.644–0.819) | 0.871 (0.848–0.891) |
| Gemma 2 27B + FSL | 0.865 (0.807–0.921) | 0.953 (0.934–0.970) | 0.877 (0.829–0.918) | 0.942 (0.923–0.957) |
| Claude 3.5 Sonnet + FSL | **0.929 (0.845–0.965)** | 0.918 (0.898–0.936) | 0.831 (0.763–0.884) | 0.944 (0.925–0.962) |
| Gemma 2 2B + SFT | 0.813 (0.746–0.887) | 0.948 (0.927–0.965) | 0.842 (0.757–0.913) | 0.928 (0.909–0.944) |
| Gemma 2 27B + QLoRA SFT | 0.921 (0.863–0.975) | **0.969 (0.953–0.981)** | **0.906 (0.860–0.944)** | **0.971 (0.956–0.983)** |

FSL, few-shot learning; SFT, supervised fine-tuning.

## Supplementary Table S4. Inter-rater agreement among the three physicians

| **Measure** | **Value** |
| --- | --- |
| Binary symptom flags assessed, n | 22 |
| Pooled Fleiss’ κ (symptom flags) | 0.31 |
| Pooled observed agreement | 0.939 |
| Mean per-item Fleiss’ κ | 0.31 |
| Mean per-item unanimous (3/3) agreement | 0.911 |
| Body temperature, all-three exact-match agreement | 0.718 |
| Vaccination description, all-three exact-match agreement | 0.995 |

Inter-rater agreement among the three physicians on the 216-note pilot set (per-item Fleiss' κ and observed agreement) for the reference standard used to extract infectious-disease information. For several low-prevalence items, observed agreement is high while κ is near zero (the κ paradox), so κ understates reliability for rare findings; the low κ for fever instead reflects a pre-harmonization difference in the physicians' operational definition of fever when no temperature was recorded, which was resolved by consensus and applied uniformly to the finalized reference standard.

## Supplementary Table S5. Comparison of annotated versus unannotated clinical notes by patient demographics and clinic

| **Group** | **Notes** | **Patients** | **Age, mean (SD)** | **Age, median** | **Male %** |
| --- | --- | --- | --- | --- | --- |
| Annotated (analyzed) | 773 (766 visits) | 526 | 18.1 (16.0) | 15 | 54.7 |
| Unannotated (excluded) | 1,037 visits | 512 | 12.3 (18.9) | 3 | 52.7 |

Tests: age, Mann–Whitney p<0.001; sex, χ² p=0.44; clinic distribution, χ² p<0.001 (the excluded notes came disproportionately from two clinics that contributed almost no analyzable notes; see Supplementary Table S6 for the analyzed-set clinic composition).

## Supplementary Table S6. Characteristics of the five participating outpatient clinics

| **Clinic (inst_id)** | **Notes** | **Patients** | **Age, mean** | **Age, median** | **Male %** | **COVID-19 mention, %** |
| --- | --- | --- | --- | --- | --- | --- |
| COV04 | 277 | 219 | 16.9 | 17.0 | 66.1 | 97.8 |
| COV01 | 216 | 156 | 35.6 | 37.0 | 46.8 | 61.1 |
| COV05 | 188 | 101 | 7.5 | 7.0 | 43.6 | 88.3 |
| COV03 | 84 | 49 | 0.8 | 1.0 | 63.1 | 58.3 |
| COV02 | 1 | 1 | 17.0 | 17.0 | 0.0 | 100.0 |

## Supplementary Table S7. Extraction performance (macro-averaged F1) stratified by primary diagnosis

| **Stratum** | **n** | **Rule-based** | **Claude 3.5 Sonnet + FSL** | **Gemma 2 27B + QLoRA SFT** |
| --- | --- | --- | --- | --- |
| COVID-19 | 120 | 0.702 | 0.886 | 0.900 |
| Non-COVID-19 | 31 | 0.605 | 0.735 | 0.893 |
| All test (reference) | 151 | 0.685 | 0.875 | 0.906 |

## Supplementary Table S8. Reference-standard reliability sensitivity analysis: extraction performance (macro-averaged F1) on triple-annotated versus single-annotated evaluation notes

| **Model** | **Triple-annotated F1 (n=45)** | **Single-annotated F1 (n=106)** | **Δ (triple − single)** |
| --- | --- | --- | --- |
| Rule-based | 0.784 | 0.688 | +0.096 |
| Gemma 2 2B + FSL | 0.636 | 0.641 | −0.005 |
| Gemma 2 27B + FSL | 0.799 | 0.892 | −0.093 |
| Claude 3.5 Sonnet + FSL | 0.797 | 0.904 | −0.108 |
| Gemma 2 2B + SFT | 0.761 | 0.814 | −0.053 |
| Gemma 2 27B + QLoRA SFT | 0.958 | 0.897 | +0.062 |

Values are matched-group macro-averaged F1 scores, averaged over the extraction categories with at least one positive case in both strata (matched-group comparison), computed with the same estimator as the main analysis. Between-stratum differences vary in direction across models and are not systematically in favor of the triple-annotated notes; the larger per-model differences reflect sampling variability in the small triple-annotated subgroup (n=45) rather than a reliability deficit of single annotation, supporting the robustness of the predominantly single-annotated reference standard.
